# Incidence & Risk Factors of Postoperative Delirium After Spinal Surgery in Older Patients

**DOI:** 10.1038/s41598-020-66276-3

**Published:** 2020-06-08

**Authors:** Taewook Kang, Si Young Park, Jin Hyeok Lee, Soon Hyuck Lee, Jong Hoon Park, Seul Ki Kim, Seung Woo Suh

**Affiliations:** Department of Orthopaedics, Korea University College of Medicine, Anam Hospital, Seoul, Korea

**Keywords:** Nutritional supplements, Risk factors

## Abstract

Although postoperative delirium is a common complication in older patients, few papers have described risk factors after of spinal surgery. The purpose of this study was to analyze various perioperative risk factors for delirium after spinal surgery in older patients. This study was performed on retrospective data collection with prospective design. We analyzed 138 patients over 65 years of age who underwent spinal surgery. Preoperative factors were cognitive function (Mini-Mental State Examination-Korean (MMSE-K) and the Korean version of the Delirium Rating Scale-Revised-98 (K-DRS 98)), age, sex, type of admission, American Society of Anesthesiologist classification, metabolic equivalents, laboratory findings, visual analog scale, and Oswestry Disability Index. Intraoperative factors were operation time, blood loss, and type of procedure. Postoperative factors were blood transfusion and type of postoperative pain control. Postoperative delirium developed in 25 patients (18.16%). Patients were divided into two groups: Group with delirium (group A) and group without delirium (group B). MMSE-K scores in Group A were significantly lower than in Group B (p < 0.001). K-DRS 98 scores were significantly higher in Group A than Group B (p < 0.001). The operation time was longer in Group A than Group B (p = 0.059). On multivariate regression analysis, the odds ratio of K-DRS 98 was 2.43 (p = 0.010). After correction for the interaction between age and MMSE-K, patients younger than 73 years old had a significantly lower incidence of delirium with higher MMSE-K score (p = 0.0014). Older age, low level of preoperative cognitive function, long duration of surgery, and transfusion were important risk factors of postoperative delirium after spinal surgery. It is important to recognize perioperative risk factors and manage appropriately.

## Introduction

Delirium is defined as a disturbance in attention, awareness, and cognition which develops over a short period of time with a fluctuating course^1^. Postoperative delirium is a common complication of surgical procedures in older patients, observed in 3.3 to 77% of cases following a variety of surgeries^2,3^. It causes difficulty in postoperative care, prolonging the hospitalization period and complicating early gait and rehabilitation after surgery, resulting in functional deterioration^4,5^. Postoperative delirium can also cause stress to patients, family members, and health care workers, and has been associated with higher mortality and additional complications^6^.

Postoperative delirium has many risk factors and is multifactorial, and identifying the possible risk factors may be helpful for its prevention^6–12^. Preoperative factors are older age, drug and alcohol abuse, bad nutrition status or low functional status, central nervous system disorder, diabetes, anemia, history of previous surgery, mental status and cognitive function^2,8,13–15^. Intraoperative factors are type of surgery, type of anesthesia, duration of operation, and blood loss^16–18^. Postoperative factors are laboratory data, amount of blood transfusion, and opioid analgesic drug use^7,17^. Patients with these risk factors are considered prone to postoperative delirium.

Postoperative delirium is a common complication after spinal surgery, and it is a complex issue involving multiple factors. Increasingly, older patients at high risk of developing postoperative delirium undergo spinal surgery. However, there is currently insufficient understanding of postoperative delirium in patients undergoing spinal surgery, and there is still a lack of clear evidence in prevention and treatment. Compared with postoperative delirium of other types of surgeries, the risk factors following spinal surgery have not been fully clarified. The individual, social, and financial burden of postoperative delirium are so great that identifying patients at risk is important for prevention and treatment to improve patient outcomes. In this study, we retrospectively analyzed the incidence and determined probable perioperative risk factors of postoperative delirium after spinal surgery in older patients.

## Materials and Methods

### Study design and subjects

This study was approved by the Institutional Review Board of Korea University Anam Hospital. Informed consent was waived because this study reviewed pre-existing data, which was approved by the Institutional Review Board of Korea University Anam Hospital. All methods were performed in accordance with the relevant guidelines and regulations.

We retrospectively reviewed the records of 138 patients (55 males and 83 females) who underwent spinal surgery in our institute between March 2016 and July 2017. Their ages ranged from 65 to 85 years, with a mean of 73.2 years. None of the patients had delirium or a history of delirium prior to surgery. Thirty-two patients had undergone operation in the cervical spine and 106 in the lumbar spine (86 patients with decompression only, 20 patients with decompression and fusion). All operations were performed by one senior spine surgeon. Atropine sulfate and hydroxyzine was administered as premedication 0.5–1 hours before surgery, and all patients received general anesthesia.

Preoperative evaluations, including type of admission (elective or emergency), American Society of Anesthesiologist (ASA) classification, metabolic equivalents (METs), preoperative level of hemoglobin (pre-Hb), visual analog scale (VAS) score, and Oswestry Disability Index (ODI) were investigated. METs were divided into 2 categories: <4 METs and ≥4 METs. For assessment of cognitive functions, all patients underwent two tests preoperatively within one day before surgery: the Mini-Mental State Examination-Korean (MMSE-K) and the Korean version of the Delirium Rating Scale-Revised-98 (K-DRS 98). ODI was investigated only in patients who underwent lumbar surgery. We evaluated operation time, blood loss, and type of procedure from the operative report for investigation of intraoperative factors. As postoperative factors, blood transfusion and type of pain control were also evaluated in all patients. We divided blood transfusion into three categories: No transfusion, intraoperative transfusion, and postoperative transfusion (not by amount of transfusion). In addition, type of pain control was divided into four categories: opioid only, non-opioid only, opioid & non-opioid, and no medication.

The presence of delirium was evaluated by consultation with the Department of Psychiatry based on the *Diagnostic and Statistical Manual of Mental Disorder* (DSM-V). The diagnostic criteria included five features: A: A disturbance in attention and awareness; B: Disturbance that develops over a short period of time, represents a change from baseline attention and awareness, and tends to fluctuate in severity during the course of a day; C: An additional disturbance in cognition; D: The disturbances in Criteria A and C are not better explained by another preexisting, established, or evolving neurocognitive disorder and do not occur in the context of a severely reduced level of arousal, such as coma; E: There is evidence from the history, physical examination, or laboratory findings that the disturbance is a direct physiological consequence of another medical condition, substance intoxication or withdrawal, or exposure to a toxin, or is due to multiple etiologies. When patients had delirious symptoms, such as disorientation, memory impairment, perceptual disturbances, psychomotor disturbances, emotional disturbances, and disturbance of the sleep-wake cycle, we consulted the psychiatrist and diagnosed the case as postoperative delirium.

### Statistical analysis

The patients were divided into two groups: Group with delirium (group A) and group without delirium (group B). Student’s t-test was used for statistical analysis of the difference in mean values between the two groups (age, VAS score, ODI, pre-Hb, MMSE-K, K-DRS 98, operation time, blood loss). The chi-square test for independence was used to compare sex, METs, type of procedure, and type of pain control. Fisher’s exact test was also used to compare the type of admission, ASA classification, and blood transfusion. To analyze risk factors of postoperative delirium, univariate and multivariate logistic regression analyses were used. Univariate logistic regression analysis was performed using explanatory variables of age, sex, pre-Hb, MMSE-K, K-DRS 98, operation time, blood loss, VAS score, ODI, and blood transfusion. Multivariate logistic regression analysis was performed with only factors that were considered to contribute to the risk, comprising age and sex, MMSE-K, K-DRS 98, operation time, and blood transfusion. The level of significance was set at *p* < 0.05. All statistical analyses were performed using SPSS ver. 20.0 (SPSS Inc., Chicago, IL, USA).

## Results

Postoperative delirium developed in 25 of 189 patients (18.16%), 11 males (44%) and 14 females (56%). The mean age of these patients was 73.5 ± 4.2 years. Among them, 23 patients (92%) were admitted to the hospital for elective surgery, and 2 (8%) were admitted through the emergency department. Twenty-two patients (88%) were ASA class II (patients with mild systemic disease), 3 patients (12%) were ASA class III (patients with severe systemic disease), and none were ASA class I.

### Preoperative risk factors

Preoperative data were compared between the two groups (Table 1). There was no statistical difference in age (73.5 ± 4.2 vs. 73.2 ± 4.8 years, p = 0.766), sex (F/M: 14/11 vs. 69/44, p = 0.640), type of admission (emergency: 2 vs. 7, elective 23 vs. 106, p = 0.666), ASA classification (1: 0 vs. 1, 2: 22 vs. 98, 3: 3 vs. 14, p > 0.999), METs (<4: 10 vs. 33, ≧4: 15 vs. 80, p = 0.292), VAS score (5.9 vs. 6.1, p = 0.478), or ODI (29.1 vs. 31.7, p = 0.215). Also, pre-Hb was not statistically different between the two groups (12.8 ± 1.3 vs. 13.0 ± 1.8 mg/dl, p = 0.469). However, preoperative MMSE-K score in group A was significantly lower than that in group B (28.1 ± 1.2 vs. 29.7 ± 0.9, p < 0.001), and K-DRS 98 score was significantly higher in group A than group B (1.9 ± 1.4 vs. 0.4 ± 1.0, p < 0.001).

**Table 1:** Comparison of preoperative data between the delirium group (group A) and non-delirium group (group B).

	Total	Group A	Group B	p-value
	(n = 138)	(n = 25)	(n = 113)	
Age, years*	73.2 (4.7)	73.5 (4.2)	73.2 (4.8)	0.766 (a)
***Male***	55 (39.9)	11 (44.0)	44 (38.9)	0.640 (b)
***Type of Admission***				0.666 (c)
Emergency	9 (6.5)	2 (8.0)	7 (6.2)	
Elective	129 (93.5)	23 (92.0)	106 (93.8)	
***ASA class***				>0.999 (c)
1	1 (0.7)	0 (0)	1 (0.9)	
2	120 (87.0)	22 (88.0)	98 (86.7)	
3	17 (12.3)	3 (12.0)	14 (12.4)	
***METs***				0.292 (b)
<4	43 (31.2)	10 (40.0)	33 (29.2)	
≧4	95 (68.8)	15 (60.0)	80 (70.8)	
Pre-Hb*	13.0 (1.7)	12.8 (1.3)	13.0 (1.8)	0.469 (a)
VAS*	6.1 (1.67)	5.9 (1.7)	6.1 (1.7)	0.478 (a)
ODI*	31.3 (7.84)	29.1 (5.6)	31.7 (8.2)	0.215 (a)
MMSE-K*	29.4 (1.11)	28.1 (1.2)	29.7 (0.9)	<0.001 (a)
K-DRS 98*	0.6 (1.2)	1.9 (1.4)	0.4 (1.0)	<0.001 (a)
Operation time*	154.6 (88.8)	185.8 (106.8)	147.7 (83.3)	0.052 (a)
Blood loss*	185.7 (157.8)	179.2 (123.3)	187.1 (164.6)	0.825 (a)
***Type of procedure***				0.289 (b)
Cervical	32 (23.2)	4 (16.0)	28 (24.8)	
Lumbar decompression	86 (62.3)	19 (76.0)	67 (59.3)	
Lumbar fusion	20 (14.5)	2 (8.0)	18 (15.9)	
***Blood transfusion***				0.254 (c)
No transfusion	99 (71.0)	15 (60.0)	84 (73.5)	
Intra-operative	13 (9.4)	4 (16.0)	9 (8.0)	
Post-operative	26 (18.8)	6 (24.0)	20 (17.7)	
***Medication***				0.504 (c)
No medication	17 (12.3)	1 (4.0)	16 (14.2)	
Opioid only	42 (30.4)	9 (36.0)	33 (29.2)	
Non-opioid only	18 (13.0)	4 (16.0)	14 (12.4)	
Both	61 (43.5)	11 (44.0)	50 (44.3)	

Note. Continuous variables(*) were presented as mean (SD) and categorical variables were summarized as frequency (valid percentage).

ASA class: American Society of Anesthesiologist classification, METs: Metabolic equivalents, Pre-Hb: preoperative level of hemoglobin, VAS: Visual Assessment Score, ODI: Oswestry Disability Index, MMSE-K: Mini-Mental State Examination – Korean, K-DRS 98: Korean version of the Delirium Rating Scale-Revised-98.

(a) p-value by Student’s t-test.

(b) p-value by Chi-square test.

(c) p-value by Fisher’s exact test.

### Intraoperative risk factors

There was no statistical difference in intraoperative factors (Table 1). Operation time of group A was longer than that of group B (185.8 ± 106.8 vs. 147.7 ± 83.3 minutes, p = 0.052), but the difference was not statistically significant. The amount of blood loss was not significantly different between the two groups (179.2 vs. 187.1 ml, p = 0.825). Type of procedure was also not significantly different (cervical: 4 vs. 28, lumbar decompression: 19 vs. 67, lumbar fusion: 2 vs. 18, p = 0.289).

### Postoperative risk factors

Postoperatively, blood transfusion and type of pain control were not significantly different in their respective categories (p = 0.254, 0.504, respectively) (Table 1). Ten patients in group A (40%) and 29 patients in group B (26.6%) had a transfusion in the intraoperative or postoperative phase. Twenty patients in group A (80%) and 83 patients in group B (73.5%) were given opioids in the postoperative phase.

### Logistic regression analysis

On univariate logistic regression analysis, preoperative MMSE-K and K-DRS 98 scores were significantly related to postoperative delirium; specifically, lower MMSE-K score (odds ratio: 0.34, p < 0.001) and higher K-DRS 98 score (odds ratio: 2.34, p < 0.001) increased the risk of delirium (Table 2). Intra- and post-operative blood transfusion also increased the risk of postoperative delirium (odds ratio: 2.46, p = 0.327/odds ratio:1.66, p = 0.921). The risk of delirium was also higher in older patients, but not significantly so (odds ratio: 1.01, p = 0.798).

**Table 2:** Univariate logistic regression analyses of risk factors for postoperative delirium.

Variables	Odds Ratio (95% CI)		p-value
Age	1.01	(0.92, 1.11)	0.798
Male	1.21	(0.51, 2.92)	0.664
Pre-Hb	0.91	(0.70, 1.17)	0.465
MMSE-K	0.34	(0.22, 0.51)	<0.001
K-DRS 98	2.34	(1.63, 3.28)	<0.001
Operation time	1.00	(1.00, 1.01)	0.059
Blood loss	1.00	(1.00, 1.00)	0.787
VAS	0.91	(0.71, 1.18)	0.490
ODI	0.96	(0.89, 1.03)	0.216
Blood transfusion			
No transfusion	1[Reference]		
Intra-operative	2.46	(0.67, 9.02)	0.327
Post-operative	1.66	(0.57, 4.82)	0.921

Pre-Hb: preoperative level of hemoglobin, MMSE-K: Mini-Mental State Examination – Korean, K-DRS 98: Korean version of the Delirium Rating Scale-Revised-98, VAS: Visual Assessment Score, ODI: Oswestry Disability Index, CI: confidence level.

On multivariate logistic regression analysis, there was a decreased risk with age (odds ratio: 0.80, p = 0.93). To investigate the interactions between age and other variables, multivariate logistic regression analysis was repeated using age and each variable. There was a significant interaction between age and MMSE-K score (p = 0.0014) (Table 3). When other variables are corrected, including the interaction of age and MMSE-K, the higher K-DRS-98 score significantly increased the risk of the incidence of delirium. Patients younger than 73 years old had a significantly lower incidence of delirium with higher MMSE-K score, one-point increase in MMSE-K score was found to reduce the risk of delirium by 87%. This effect was greater in patients under 73 years old than patients older than 73 years.

**Table 3:** Multivariate logistic regression analyses of risk factors for postoperative delirium.

Variables	Odds Ratio (95% CI)		p-value
Male	2.09	(0.52, 8.42)	0.300
K-DRS 98	2.43	(1.24, 4.77)	0.010
Operation time	1.00	(0.99, 1.01)	0.707
Blood transfusion			
No transfusion	1[Reference]		
Intra-operative	1.00	(0.06, 17.81)	0.999
Post-operative	1.58	(0.26, 9.41)	0.618
MMSE-K			0.014*
At age ≥ 73	0.73	(0.32, 1.64)	
At age < 73	0.13	(0.03, 0.51)	

*p-value for interaction between age and MMSE-K.

MMSE-K: Mini-Mental State Examination – Korean, K-DRS 98: Korean version of the Delirium Rating Scale-Revised-98, CI: confidence level.

## Discussion

In our study, the overall prevalence of postoperative delirium after spinal surgery was 18.16%. We found that older age, low level of preoperative cognitive function (lower MMSE-K, higher K-DRS 98), long duration of surgery, and transfusion were important risk factors of postoperative delirium after spinal surgery. Both modifiable and non-modifiable risk factors contributed to risk for postoperative delirium after spinal surgery. However, duration of surgery and transfusion were the only two modifiable risk factors, and all other risk factors were non-modifiable.

The incidence of postoperative delirium after spinal surgery was from 0.49% to 21% in previous reports^7,10,19–21^. In our study, the incidence of postoperative delirium was 18.16% and comparable with other studies. Kobayashi *et al*. reported the incidence of postoperative delirium to be 0.49% after lumbar spinal surgeries and significantly more occurence in older patients^20^. Fineberg *et al*. reported the incidence of postoperative delirium of 0.84% after lumbar spinal surgeries^19^. Kawaguchi *et al*. reported the incidence of postoperative delirium of 12.5% in patients older than 70 years^7^. Morino *et al*. reported the incidence of postoperative delirium of 11.1% after spinal surgery^10^.

There are several reports of risk factors for postoperative delirium, and the risk is multifactorial^22^. Predisposing factors include older age, cognitive impairment, alcohol/drug abuse and dependence, psychiatric comorbidity, sensory impairment, and dehydration/malnutrition. In addition, functional dependence is one of most important predisposing factors; use of stretchers or wheelchairs at admission was significantly higher in patients with delirium than those without delirium. This may indicate that patients with postoperative delirium had poorer preoperative physical condition. Patients who undergo spinal surgery complain about the impaired activities of daily life due to pain in the trunk and extremities. Therefore, patients undergoing spinal surgery tend to be prone to postoperative delirium.

Age is a major factor affecting the onset of delirium^23^. Our study indicated that age older than 70 years were associated with an increased risk of delirium. The higher incidence of postoperative delirium among the older patients may be associated with increased comorbidities, decreased physical activity, decreased brain volume, and decreased cerebral neurotransmitters production^24^. The narrowing of vessels due to vascular disease which is associated with aging decreases oxygen supply to the brain and promote the development of postoperative cerebral dysfunction^24^.

If postoperative delirium occurs, the length of hospital stay is prolonged, and the likelihood of medical complications is increased^25,26^. It is reported that the postoperative outcomes of delirious patients at discharge are worse than those of non-delirious patients because of difficulties in early ambulation and rehabilitation exercises. Although delirium can be caused by a single factor, the incidence of delirium increases as the number of risk factors increases^27,28^. It is important to prevent delirium through a multifaceted approach to reduce risk factors. Inouye reported that delirium-related risk factors could be improved in older patients to reduce the incidence and duration of delirium^17^.

The risk factors described above should be assessed at hospital admission. There are non-modifiable and modifiable risk factors. In our study, age and preoperative cognitive function were non-modifiable factors, and operation time and transfusion were modifiable factors. Active interventions were needed to correct risk factors before and after surgery^17,27,28^. We should try to shorten the duration of surgery and reduce rate of transfusion, as these are modifiable factors. Even with non-modifiable factors, recognizing, preparing, and predicting the risk factors could help preventing postoperative delirium and providing appropriate treatment. Encouraging postoperative ambulation and preventing other possible medical complications after surgery are thought to reduce delirium. For patients at high risk of postoperative delirium, supportive postoperative care is needed. In patients with postoperative delirium, well-organized wards, emotional support from family members, appropriate stimuli, and use of fewer medications can be helpful in the treatment of delirium and can reduce the duration of delirium^17,27^.

This study has several limitations. First, it was a retrospective study based on a data review that could not evaluate of severity and details of delirium. Second, diagnosis of delirium was based on consultation with the Department of Psychiatry, so details of the delirium were not available. Third, the risk for delirium is multifactorial, and there are many other potential risk factors that we did not assess. Other risk factors that may affect postoperative delirium and how they interact need to be explored. Understanding modifiable and non-modifiable risk factors can improve prevention and management of postoperative delirium. The risk of postoperative delirium can be reduced with careful attention to perioperative risk factors^29^.

## Conclusions

Older age and low preoperative cognitive function were the most important risk factors of postoperative delirium after spine surgery. In addition, longer duration of surgery and transfusion can affect postoperative delirium. Surgeons should consider these risk factors in patients at high risk for postoperative delirium.

## References

[1] Association, A. P. *Diagnostic and statistical manual of mental disorders (DSM-5®)*. (American Psychiatric Pub, 2013). 10.1590/s2317-17822013000200017 24413388

[2] Schmitt EM (2012). Novel risk markers and long-term outcomes of delirium: the successful aging after elective surgery (SAGES) study design and methods. J. Am. Med. Dir. Assoc..

[3] Scott JE, Mathias JL, Kneebone AC (2015). Incidence of delirium following total joint replacement in older adults: a meta-analysis. Gen. Hosp. Psychiatry.

[4] Brown TM, Boyle MF (2002). Delirium. BMJ.

[5] Gleason LJ (2015). Effect of Delirium and Other Major Complications on Outcomes After Elective Surgery in Older Adults. JAMA Surg..

[6] Marcantonio ER, Goldman L, Orav EJ, Cook EF, Lee TH (1998). The association of intraoperative factors with the development of postoperative delirium. Am. J. Med..

[7] Kawaguchi Y (2006). Postoperative delirium in spine surgery. Spine J..

[8] Gao R, Yang ZZ, Li M, Shi ZC, Fu Q (2008). Probable risk factors for postoperative delirium in patients undergoing spinal surgery. Eur. Spine J..

[9] Ushida T (2009). Incidence and risk factors of postoperative delirium in cervical spine surgery. Spine.

[10] Morino T (2018). Risk Factors for Delirium after Spine Surgery: An Age-Matched Analysis. Asian Spine J..

[11] Weinstein, S. M. *et al*. Postoperative delirium in total knee and hip arthroplasty patients: a study of perioperative modifiable risk factors. *Br J Anaesth* **120** (2018). 10.1016/j.bja.2017.12.046 29661417

[12] Song KJ, Ko JH, Kwon TY, Choi BW (2019). Etiology and Related Factors of Postoperative Delirium in Orthopedic Surgery. Clin. Orthop. Surg..

[13] Robinson TN, Wu DS, Pointer LF, Dunn CL, Moss M (2012). Preoperative cognitive dysfunction is related to adverse postoperative outcomes in the elderly. J. Am. Coll. Surg..

[14] Wilson CA, Roffey DM, Chow D, Alkherayf F, Wai EK (2016). A systematic review of preoperative predictors for postoperative clinical outcomes following lumbar discectomy. Spine J..

[15] Adogwa O (2018). Association between baseline cognitive impairment and postoperative delirium in elderly patients undergoing surgery for adult spinal deformity. J. Neurosurg. Spine.

[16] Bucerius J (2004). Predictors of delirium after cardiac surgery delirium: effect of beating-heart (off-pump) surgery. J. Thorac. Cardiovasc. Surg..

[17] Inouye SK (2006). Delirium in older persons. N. Engl. J. Med..

[18] Schneider F (2002). Risk factors for postoperative delirium in vascular surgery. Gen. Hosp. Psychiatry.

[19] Fineberg SJ (2013). Incidence and risk factors for postoperative delirium after lumbar spine surgery. Spine.

[20] Kobayashi K (2017). Risk Factors for Delirium After Spine Surgery in Extremely Elderly Patients Aged 80 Years or Older and Review of the Literature: Japan Association of Spine Surgeons with Ambition Multicenter Study. Glob. Spine J..

[21] Susano MJ (2019). Retrospective Analysis of Perioperative Variables Associated With Postoperative Delirium and Other Adverse Outcomes in Older Patients After Spine Surgery. J. Neurosurg. Anesthesiol..

[22] Inouye SK, Charpentier PA (1996). Precipitating factors for delirium in hospitalized elderly persons. Predictive model and interrelationship with baseline vulnerability. JAMA.

[23] Chung KS, Lee JK, Park JS, Choi CH (2015). Risk factors of delirium in patients undergoing total knee arthroplasty. Arch. Gerontol. Geriatr..

[24] Shi C, Yang C, Gao R, Yuan W (2015). Risk Factors for Delirium After Spinal Surgery: A Meta-Analysis. World Neurosurg..

[25] Zywiel MG (2015). Health economic implications of perioperative delirium in older patients after surgery for a fragility hip fracture. J. Bone Jt. Surg. Am..

[26] Lee JK, Park YS (2010). Delirium after spinal surgery in Korean population. Spine.

[27] Robertson BD, Robertson TJ (2006). Postoperative delirium after hip fracture. J. Bone Jt. Surg. Am..

[28] Marcantonio E, Ta T, Duthie E, Resnick NM (2002). Delirium severity and psychomotor types: their relationship with outcomes after hip fracture repair. J. Am. Geriatr. Soc..

[29] Amador LF, Goodwin JS (2005). Postoperative delirium in the older patient. J. Am. Coll. Surg..

